# Reply to: Revisiting the origin of octoploid strawberry

**DOI:** 10.1038/s41588-019-0544-2

**Published:** 2019-12-16

**Authors:** Patrick P. Edger, Michael R. McKain, Alan E. Yocca, Steven J. Knapp, Qin Qiao, Ticao Zhang

**Affiliations:** 1 Department of Horticulture, Michigan State University, East Lansing, MI USA; 2 Ecology, Evolutionary Biology and Behavior, Michigan State University, East Lansing, MI USA; 3 Department of Biological Sciences, University of Alabama, Tuscaloosa, AL USA; 4 Department of Plant Biology, Michigan State University, East Lansing, MI USA; 5 Department of Plant Sciences, University of California–Davis, Davis, CA USA; 6 School of Agriculture, Yunnan University, Kunming, China; 7 College of Chinese Material Medica, Yunnan University of Chinese Medicine, Kunming, China

**Keywords:** Plant sciences, Computational biology and bioinformatics

replying to A. Liston et al. *Nature Genetics* 10.1038/s41588-019-0543-3 (2019)

The origin of octoploid strawberry has been the focus of several phylogenetic studies over the past decade (for example, refs. ^1–3^). Our previous study, using the octoploid genome and transcriptomes of every extant diploid *Fragaria* species, provided support for four species (*Fragaria* *vesca*, *Fragaria* *iinumae,* *Fragaria* *viridis* and *Fragaria* *nipponica*) as the closest extant relatives of the diploids that contributed to the origin of octoploid strawberry^4^. In a response paper^5^, Liston et al. stated “that only two extand diploids were progenitors” with one subgenome being contributed by *F. vesca* and three by *F. iinumae–like* ancestors. Our reanalysis of the transcriptome data and comparative genomic analyses of a chromosome-scale *F. iinumae* genome support our previous model for the origin of octoploid strawberry^4^.

Liston et al.^5^ raised a concern regarding one of the steps in the phylogenetic analysis of the subgenome tree-searching algorithm (PhyDS) tool we developed to identify extant relatives of diploid progenitors of allopolyploids. Specifically, they argue that we may have incorrectly identified *F. viridis* and *F. nipponica* as extant relatives because in-paralogs were excluded from our previous phylogenetic analysis^4^. Our reanalysis of the data using PhyDS, now including in-paralogs, yielded results consistent with those presented in our previous study (Fig. 1; Supplementary Information and Supplementary Dataset 1). Furthermore, their alternative model for the origin of octoploid strawberry (1× *F. vesca*–like and 3× *F. iinumae*–like subgenomes) is not supported by comparative genomic analyses of a new chromosome-scale *F. iinumae* genome (Fig. 2).

**Fig. 1:**
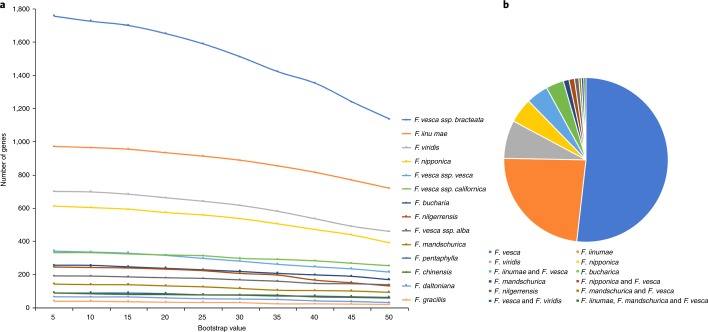
Phylogenetic analyses. **a**, Number of genes from species identified as being sister to a homoeolog from the octoploid genome, by using PhyDS with bootstrap support value (BSV) cutoffs. Based on previous results^4^. **b**, Reanalysis of the data, including in-paralogs and BSV_50_ cutoff, identified the same progenitor species. The prevalence and biased patterns of homoeologous exchanges between subgenomes resulted in the dominant *F. vesca* subgenome replacing a greater number of corresponding regions in each of the recessive subgenomes^4^. Thus, a greater number of genes from the dominant *F. vesca* subgenome were identified, with the *F. iinumae*–like subgenome being second.

**Fig. 2:**
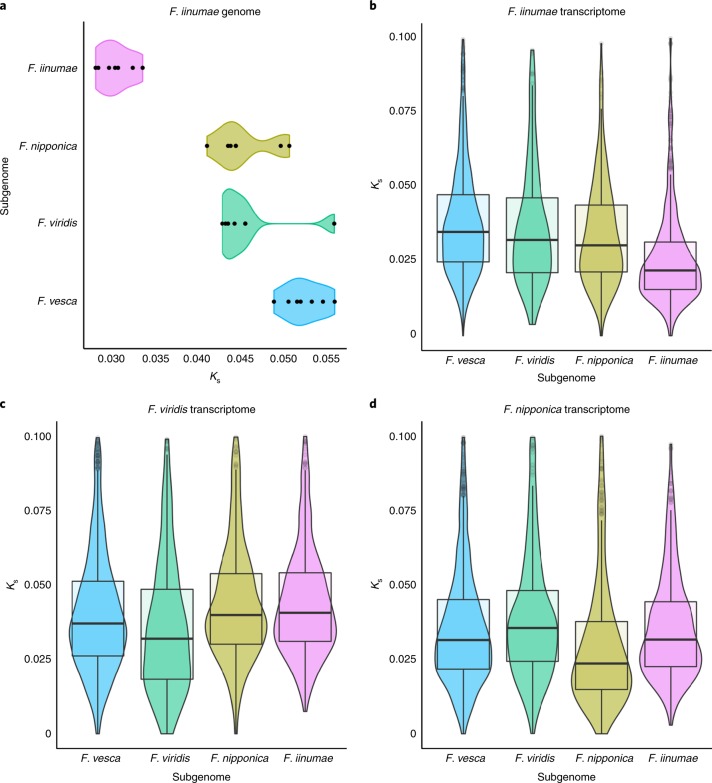
Divergence of *K*_s_ rates among subgenomes. **a**, Synonymous substitution divergence for all syntenic genes between the *F. iinumae* and *Fragaria* × *ananassa* genomes^4^. The median *K*_s_ divergence values for the seven chromosomes previously assigned to each progenitor species are plotted. The *F. iinumae* and *F. vesca* subgenomes exhibit the lowest and highest *K*_s_ divergence, respectively. **b**–**d**, *K*_s_ analysis of *F. iinumae* (**b**), *F. viridis* (**c**) and *F. nipponica* (**d**) transcriptomes against the phylogenetically supported homoeolog in the octoploid genome. The *K*_s_ distributions of *F. viridis* and *F. nipponica* transcriptomes are both unique and distinct from that of *F. iinumae*.

Phylogenetic analysis of the subgenome tree-searching algorithm searched a set of gene trees to identify sequences most closely related to a set of user-provided paralogs (or homoeologs in polyploids). Homoeologs are orthologous genes that were brought back into the same nucleus by allopolyploidization^6^. For our analyses, we used syntenic (that is, positionally conserved) homoeologs that were present on all subgenomes in octoploid strawberry. Gene trees were estimated using RAxML^7^ based on orthologs identified using established orthogrouping approaches^8^ applied to de novo assembled transcriptomes for each diploid *Fragaria* species^4^. PhyDS performs a relatively simple and straightforward analysis of gene trees. First, it identifies the user-provided paralog present in a gene tree and then moves to the direct ancestral node of the paralog. Second, PhyDS then returns to the user the direct descendants (that is, sequence identities including the paralog) of that ancestral node with its bootstrap support value (Fig. 1).

We have two major concerns regarding the methods used in refs. ^2,5^. First, phylogenetic analyses aimed at estimation of species relationships are reliant first on correct identification of orthologs^9^. These authors used a sequence similarity-based approach to identify putative orthologs that has relatively high error rates^10^. Furthermore, pangenome studies have shown that up to one-half of gene content exhibits presence–absence variation at the species level in plants^11^. In other words, many genes are individual- or population-specific. Thus, many of the putative ortholog predictions in their studies may be inaccurate. Second, Liston et al.^5^ performed analyses of 100-kb windows across each of the seven base chromosomes. This could be problematic because chromosomal regions from one parental species can be replaced with chromosomal regions from the other parental species during meiosis in polyploids (referred to as homoeologous exchanges^12^). Homoeologous exchanges can range in size from large megabase-sized regions to single genes (see a recent review on its impact on subgenome assignment in ref. ^13^). We identifed extensive homoeologous exchanges throughout the octoploid strawberry genome^4^. Thus, the 100-kb windows Liston et al. used consist of genes with different evolutionary histories reflecting each of the different progenitor species. This could result in inaccurate estimates of species relationships.

Here we present a chromosome-scale genome of *F. iinumae* with a scaffold minimum scaffold length needed to cover 50% of the genome of 33.98 Mb and 23,665 protein-coding genes (see Supplementary Information). This genome was used to calculate the synonymous substitution (*K*_s_) divergence between *F. iinumae* to each of the four subgenomes (Fig. 2a). This revealed that only one of the subgenomes of octoploid strawberry is *F. iinumae*–like, which does not support the model presented by Liston et al.^5^ that the origin of octoploid strawberry involved three *F. iinumae*–like and one *F. vesca*–like progenitor species. Instead, these results are consistent with our phylogenetic estimates supporting more than two diploid progenitors (Fig. 2b–d). The *F. viridis* (Fig. 2c) and *F. nipponica* (Fig. 2d) subgenomes are not *F. iinumae*–like.

Our new phylogenetic analyses support four distinct progenitor species, which is consistent with our previous results^4^ and that of other groups^3^. The conflicting results obtained by Liston et al.^5^ are probably due to differences in methodology. As pointed out above, establishing gene orthology is crucial for molecular phylogenetics. Our pipeline started by identifying high-confidence syntenic 1:1 homoeologs present on each of the subgenomes. This step alone filtered out 82.1% of genes from the octoploid strawberry genome^4^. The number of genes analyzed in our study was further reduced due to absence across transcriptome data, stringent orthogroup filtering and bootstrap value filtering. In short, more data are not always better if one introduces ‘phylogenetic noise’. It is unclear to us how Liston et al.^5^ obtained high unique mapping rates (~89% alignment) across the *F. vesca* genome, which consists of ~31% transposable elements and hundreds of duplicate genes. Furthermore, many genes are species-specific based on previous pangenome studies.

As pointed out by Liston et al.^5^, incomplete lineage sorting can impact phylogenetic inferences. However, that is far more likely to impact within-species than between-species estimates. This is exactly what was observed in our study. Other *F. vesca* subspecies were identified as contributors but were present at notably lower levels than *F. viridis* and *F. nipponica* (Fig. 1a). These patterns provide further support for *F. viridis* and *F. nipponica* as extant relatives of the progenitors that contributed to the origin of the intermediate hexaploid ancestor. Lastly, we did state that *F. moschata* may be an extant relative of the intermediate hexaploid ancestor. Given the high frequency of polyploid formation in *Fragaria*^14^ and birth–death dynamics of polyploids^15^, we agree it is possible that the hexaploid ancestor may be extinct. This remains to be properly evaluated using robust phylogenetic approaches and datasets.

## Reporting Summary

Further information on research design is available in the Nature Research Reporting Summary linked to this article.

## Online content

Any methods, additional references, Nature Research reporting summaries, source data, extended data, supplementary information, acknowledgements, peer review information; details of author contributions and competing interests; and statements of data and code availability are available at 10.1038/s41588-019-0544-2.

## Supplementary information

Supplementary Information Supplementary Note and Tables 1–4

Reporting Summary

Supplementary Dataset Results from PhyDS analysis.

## Figures and Tables

**Extended Data Fig. 1:**
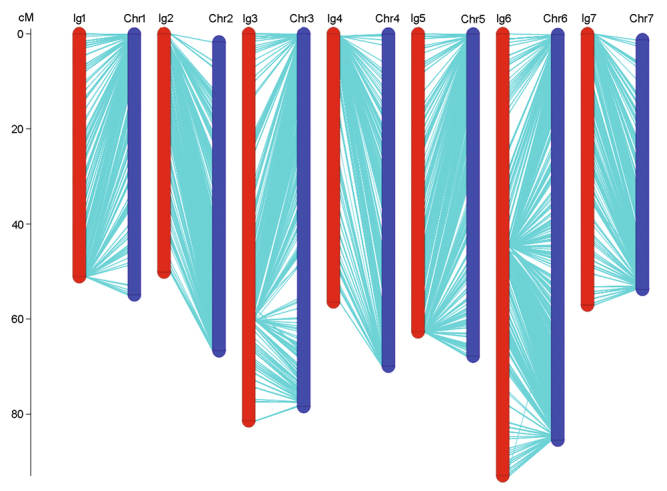
Anchoring genome sequence to the genetic map. Previously a high-density linkage map of *F. iinumae* was constructed by 4173 markers, with 3280 from the Array and 893 from genotyping by sequencing^7^. Here we anchored the contigs to this genetic map to obtain a chromosome-scale genome of *F. iinumae*.

## Data Availability

The phylogenetic trees and alignments are available on Dryad (10.5061/dryad.b2c58pc). The genome assembly and annotation files are available on the Genome Database for Rosaceae (https://www.rosaceae.org/) and NCBI GenBank under BioProjects PRJNA544784 and PRJNA508389. The raw sequence data are available in the Sequence Read Archive under the same NCBI BioProject numbers, PRJNA544784 and PRJNA508389.

## Code availability

Custom software for running PhyDS phylogenetic analyses is available on GitHub (https://github.com/mrmckain/PhyDS/).
