# Supplementary material for: Reply to: Revisiting the origin of octoploid strawberry
Source: Nat Genet. 2019 Dec 16;52(1):5–7. doi: 10.1038/s41588-019-0544-2 (PMC6960091; doi:10.1038/s41588-019-0544-2)
Supplement: Supplementary file 1 — Supplementary Note and Tables 1–4 [file 41588_2019_544_MOESM1_ESM.pdf]

nature genetics

SUPPLEMENTARY INFORMATION

https://doi.org/10.1038/s41588-019-0544-2

In the format provided by the authors and unedited.

Reply to: Revisiting the origin of octoploid strawberry

Patrick P. Edger[1,2,8]*, Michael R. McKain[3,8], Alan E. Yocca[1,4], Steven J. Knapp[5], Qin Qiao[6]* and Ticao Zhang[7]*

[1]Department of Horticulture, Michigan State University, East Lansing, MI, USA. [2]Ecology, Evolutionary Biology and Behavior, Michigan State University, East Lansing, MI, USA. [3]Department of Biological Sciences, University of Alabama, Tuscaloosa, AL, USA. [4]Department of Plant Biology, Michigan State University, East Lansing, MI, USA. [5]Department of Plant Sciences, University of California–Davis, Davis, CA, USA. [6]School of Agriculture, Yunnan University, Kunming, China. [7]College of Chinese Material Medica, Yunnan University of Chinese Medicine, Kunming, China. [8]These authors contributed equally: Patrick P. Edger, Michael R. McKain. *e-mail: edgerpat@msu.edu; qiaoqin@ynu.edu.cn; zhangticao@mail.kib.ac.cn

Supplementary Information

Reply to 'Revisiting the origin of the octoploid Strawberry'

Authors: Patrick P. Edger[1,2,*,#], Michael R. McKain[3,*], Alan E. Yocca[1,4], Steven J. Knapp[5], Qin Qiao[6,#], Ticao Zhang[12,#]

1. Department of Horticulture, Michigan State University, East Lansing, MI, USA.
2. Ecology, Evolutionary Biology and Behavior, Michigan State University, East Lansing, MI, USA
3. Department of Biological Sciences, University of Alabama, Tuscaloosa, AL, USA
4. Department of Plant Biology, Michigan State University, East Lansing, MI, USA
5. Department of Plant Sciences, University of California–Davis, Davis, California, USA
6. School of Agriculture, Yunnan University, Kunming, China
7. College of Chinese Material Medica, Yunnan University of Chinese Medicine, Kunming, China

* These authors contributed equally to this work.
# Correspondences: P.P.E. (edgerpat@msu.edu), Q.Q. (qiaoqin@ynu.edu.cn), and T.Z. (zhangticao@mail.kib.ac.cn).

Supplementary Note

1. Phylogenetic Analyses

PhyDS was modified to allow for the inclusion of in-paralogs in the analysis. These modifications also included an ignore option so the user can selectively ignore certain taxa in the search algorithm as well as inclusion of clades with more than a single taxon in results for estimation of relationships to focal paralogs. The ignore parameter allows the user more control over what PhyDS finds as an acceptable occurrence to stop the searching algorithm for a particular paralog's closest relatives. The latest version of PhyDS v. 2.1 is available at https://github.com/mrmckain/PhyDS.

Orthogroup trees were queried using the syntelog set identified in Edger et al. 2019[1] with and without ignoring other genes from *Fragaria* x *ananassa* Camarosa (--ignore Fxa option) and with a minimum bootstrap value of 50 and 80 using PhyDS v.2.1. By ignoring other *F.* x *ananassa* Camarosa genes, we only stop the PhyDS search for a relative of the target paralogs when either a) the other paralog is found or b) a different taxon is found. By not ignoring other *F.* x *ananassa* Camarosa genes, we are allowing instances where a *F.* x *ananassa* Camarosa gene that is not the target paralog's mate to stop the search. Since the syntelog set represents a highly filtered portion of the *F.* x *ananassa* Camarosa genome, we used a script derived from PUG v.2.1 to estimate all possible gene pairs from *F.* x *ananassa* Camarosa given the orthogroup trees. These gene pairs were queried in the same manner as the above. We also took the chromosomes from Edger et al. (2019) assigned to predominantly a *F. viridis*-like subgenome and a *F. nipponica*-like subgenome and filtered both the syntelog and orthogroup-

derived paralog pairs into sets based on these putative histories. These sets of paralogs were used to query the ortholog trees as above using PhyDS.

Results are available in Figure 1 and Supplementary Dataset 1. Results of all analysis coincide with the results of Edger et al. (2019). The comparison of with and without ignoring the *F.* x *ananassa* Camarosa genes in the PhyDS search allows us to identify instances where the proposed hypothesis of Liston et al. would be supported for the "C" and "D" genomes. To support the hypothesis that the "C" and "D" genomes are sister to each other and then sister to *F. iinumae*, we would expect there to be a high number of paralogs that are most closely related to another *F.* x *ananassa* Camarosa gene when we do not ignore other *F.* x *ananassa* genes in the analysis. We would also expect that a large proportion of those paralogs have *F. iinumae* as the most closely related diploid relatively when we ignore other *F.* x *ananassa* genes. Our results demonstrate that there is a large number of paralogs that find other *F.* x *ananassa* genes before they find a diploid relative across all analyses. These are the "Fxa" row in Supplementary Dataset 1. However, we do not see a large component in a clade with *F. iinumae* when we ignore the other *F.* x *ananassa* genes. Instead, there is a distribution across multiple putative diploid relatives with *F. vesca, F. iinumae, F. viridis,* and *F. nipponica* being in the largest proportions except for *Malus domestica*[2], which is likely due to these being derived from a whole genome sequence and not transcriptomes.

2. Genome Assembly and Analyses

Samples collection and Sequencing

Young leaves of *Fragaria iinumae* collected from the strawberry germplasm resources nursery in Shenyang Agricultural University were used for high-molecular weight genomic DNA extraction. An SMRTbell DNA library was then prepared and sequenced according to the manufacturer's protocols (Pacific Biosciences, CA, USA), and a 20-kb SMRTbell library was generated using a BluePippin DNA size selection instrument (Sage Science, MA, USA) with a lower size limit of 10kb. Single-molecule real-time sequencing of long reads was conducted on a PacBio Sequel platform with 9 SMRT cells which generated a total of 45.77Gb of data (Supplementary Table 1). For Illumina sequencing, paired-end (PE) libraries with insert sizes of 450bp and 250bp were constructed and sequenced on an Illumina HiSeq X Ten platform. A total of 28.42Gb of Illumina genomic data was generated (Supplementary Table 1). Illumina data were used for genome size estimation, correction of genome assembly and assembly evaluation. Genome size and heterozygosity were estimated using Illumina data and the *k*-mer statistics[3,4].

Total RNA was extracted from leave, flower and fruit organs of *F. iinumae* using the QIAGEN RNeasy Plant Mini Kit (QIAGEN, Hilden, Germany). RNA-seq libraries were then prepared using the TruSeq RNA Library Preparation Kit (Illumina, CA, USA), and paired-end sequencing with a read length of 150 bp was conducted on the HiSeq 2000 platform. The RNAseq data were used for genome annotation.

Genome assembly and quality evaluation

Genome assembly was performed on PacBio long reads using FALCON v0.3.0(GitHub, 2018. Mar 18)(Supplementary Table 1). Total genome coverage (~172X) before assembly was estimated by total bases from PacBio reads divided by the genome size (265.56 Mb) for *F. iinumae*. Error correction and preassembly were carried out with the FALCON pipeline after evaluating the outcomes of using different parameters in FALCON during the pre-assembly process. The draft genome, with a contig N50 of >10Mb, was polished with Arrow using all SMRT reads and polished using Pilon v1.22[5] using the Illumina reads (~107X coverage) with the default settings. A GC depth analysis was conducted to assess the potential contamination during sequencing and the coverage of the assembly. The completeness of the genome assembly was also evaluated using BUSCO (Benchmarking Universal Single Copy Orthologs) software[6] (Supplementary Table 2).

Anchoring genome sequence to the genetic map

Previously a high-density linkage map of *F. iinumae* was constructed by 4173 markers, with 3280 from the Array and 893 from genotyping by sequencing[7]. Here we anchored the contigs (Supplementary Table 1) to this genetic map to obtain a chromosome-scale genome of *F. iinumae* (Extended Data Figure 1).

Genome annotation

For repeat detection, four software packages, i.e., RepeatModeler[8,9] (http://www.repeatmasker.org/RepeatModeler.html), RepeatScout[10] (http://www.repeatmasker.org), Piler[11] (http://www.drive5.com/piler/), and LTR-Finder[12] (http://tlife.fudan.edu.cn/ltr_finder), were used to build a *de novo* repeat library on the basis of our assembly with the default settings. To identify known transposable elements (TEs) in the genomes, RepeatMasker[8] (http://www.repeatmasker.org) was used to screen the assembled genome against the Repbase v22.11[13] and Mips-REdat libraries[14] (Supplementary Table 3).

We constructed a *de novo* long terminal repeat retrotransposon (LTR-RT) library by scanning the assembled *F. iinumae* genome using LTRharvest[15] (-motif tgca -motifmis 1) and LTR_Finder[12] (LTR length 100-5000nt, length between two LTRs: 1000-20000nt). Homolog-based, *de novo*-based, and RNA-sequencing (RNA-seq)-based gene prediction methods were used in combination to identify the protein-coding genes in the *F. iinumae* genome assembly. For homology-based predictions, protein sequences of *Arabidopsis thaliana*, *Oryza sativa*, *Solanum lycopersicum*, *Fragaria vesca*, and *Malus domestica* were used as the references. For *de novo*-based prediction, Augustus v2.4[16], GlimmerHMM v3.0.4[17], SNAP v2006[18], GeneID v1.4[19] and Genscan[20] with default parameters were used for *de novo*-based gene prediction. All software was trained using the 1000 full-length genes from the homology-based predictions and Arabidopsis gene model before gene prediction (Supplementary Table 2 & 4). For the RNA-seq-based prediction, TransDecoder v2.0 (http://transdecoder.github.io.), GeneMarkS-T v5.1[21], and PASA v2.0.2[22] were used. Finally, the results from the three methods were integrated using EVM v1.1.1[23]. All the genes were annotated by aligning to the Nucleotide collection (NR), Swiss-Prot, Kyoto Encyclopedia of Genes and Genomes (KEGG database release 84.0). Then,

InterProScan[24] package was used to annotate the predicted genes using the InterPro (5.21–60.0) database.

Comparative Genomics

The octoploid strawberry[1] and *F. iinumae* genomes were aligned in CoGe's SynMap program with LAST[25]. The maximum distance between two matches was set to 20 genes, and the minimum number of aligned pairs was set to ten genes. Neighboring syntenic blocks were merged with 'Quota Align Merge'[26], with the maximum distance between two blocks set to 40 genes. Syntenic depth was calculated with 'Quota Align', and the ratio of coverage depth for *F. iinumae* to *F. ananassa* gene was set to 1:4. Tandemly duplicated genes were identified and filtered from CoGe outputs with a max distance of ten genes. Synonymous mutation (Ks) rates was then calculated between syntenic gene pairs within CoGe. The analysis of Ks divergence permits the rapid identification of genes and genomic regions with different evolutionary histories [27]. These analyses can be regenerated with CoGe (see URLs). Syntenic gene pairs with Ks divergence of <4 were used to calculate median Ks estimates for each chromosome. The median Ks estimates for each chromosome plotted using ggplot2 in R [28,29].

Lastly, a Ks analysis of the assembled transcriptome data [1] against the orthologs in the 'Camarosa' genome using FastKs (available on GitHub; https://github.com/mrmckain/FASTKs) was run and the genes identified with PhyDs (i.e. those with phylogenetic support) for each of the four-progenitor species are plotted in Figure 2. The distributions of *F. iinumae*, *F. nipponica*, and *F. viridis* against their respective transcriptomes are each unique. This further supports our model that four progenitors were involved in the formation of the octoploid strawberry.

References

1. Edger, P. P. *et al.* Origin and evolution of the octoploid strawberry genome. *Nat. Genet.* **51**, 541–547 (2019).

2. Daccord, N. *et al.* High-quality de novo assembly of the apple genome and methylome dynamics of early fruit development. *Nat. Genet.* **49**, 1099–1106 (2017).

3. Zhou, W.-W. *et al.* Speciation in the Rana chensinensis species complex and its relationship to the uplift of the Qinghai-Tibetan Plateau. *Mol. Ecol.* **21**, 960–973 (2012).

4. Liu, B. *et al.* Estimation of genomic characteristics by analyzing k-mer frequency in de novo genome projects. *arXiv [q-bio.GN]* (2013).

5. Walker, B. J. *et al.* Pilon: An Integrated Tool for Comprehensive Microbial Variant Detection and Genome Assembly Improvement. *PLoS One* **9**, (2014).

6. Simão, F. A., Waterhouse, R. M., Ioannidis, P., Kriventseva, E. V. & Zdobnov, E. M.

BUSCO: assessing genome assembly and annotation completeness with single-copy orthologs. *Bioinformatics* **31**, 3210–3212 (2015).

7. Mahoney, L. L. *et al.* A High-Density Linkage Map of the Ancestral Diploid Strawberry, Fragaria iinumae, Constructed with Single Nucleotide Polymorphism Markers from the IStraw90 Array and Genotyping by Sequencing. *Plant Genome* **9**, (2016).
8. Tarailo-Graovac, M. & Chen, N. Using RepeatMasker to identify repetitive elements in genomic sequences. *Curr. Protoc. Bioinformatics* **25**, 4–10 (2009).
9. Smit, A. F. A. & Hubley, R. RepeatModeler Open-1.0. 2008--2015. *Seattle, USA: Institute for Systems Biology. Available from: httpwww. repeatmasker. org, Last Accessed May* **1**, 2018 (2015).
10. Price, A. L., Jones, N. C. & Pevzner, P. A. De novo identification of repeat families in large genomes. *Bioinformatics* **21 Suppl 1**, i351–8 (2005).
11. Edgar, R. C. & Myers, E. W. PILER: identification and classification of genomic repeats. *Bioinformatics* **21 Suppl 1**, i152–8 (2005).
12. Xu, Z. & Wang, H. LTR_FINDER: an efficient tool for the prediction of full-length LTR retrotransposons. *Nucleic Acids Res.* **35**, W265–8 (2007).
13. Bao, W., Kojima, K. K. & Kohany, O. Repbase Update, a database of repetitive elements in eukaryotic genomes. *Mobile DNA* **6**, (2015).
14. Nussbaumer, T. *et al.* MIPS PlantsDB: a database framework for comparative plant genome research. *Nucleic Acids Res.* **41**, D1144–51 (2013).
15. Ellinghaus, D., Kurtz, S. & Willhoeft, U. LTRharvest, an efficient and flexible software for de novo detection of LTR retrotransposons. *BMC Bioinformatics* **9**, 18 (2008).
16. Stanke, M. & Waack, S. Gene prediction with a hidden Markov model and a new intron submodel. *Bioinformatics* **19 Suppl 2**, ii215–25 (2003).
17. Majoros, W. H., Pertea, M. & Salzberg, S. L. TigrScan and GlimmerHMM: two open source ab initio eukaryotic gene-finders. *Bioinformatics* **20**, 2878–2879 (2004).

18. Bromberg, Y. & Rost, B. SNAP: predict effect of non-synonymous polymorphisms on function. *Nucleic Acids Res.* **35**, 3823–3835 (2007).

19. Blanco, E., Parra, G. & Guigó, R. Using geneid to identify genes. *Curr. Protoc. Bioinformatics* **Chapter 4**, Unit 4.3 (2007).

20. Burge, C. & Karlin, S. Prediction of complete gene structures in human genomic DNA. *J. Mol. Biol.* **268**, 78–94 (1997).

21. Tang, S., Lomsadze, A. & Borodovsky, M. Identification of protein coding regions in RNA transcripts. *Nucleic Acids Res.* **43**, e78 (2015).

22. Campbell, M. A., Haas, B. J., Hamilton, J. P., Mount, S. M. & Buell, C. R. Comprehensive analysis of alternative splicing in rice and comparative analyses with Arabidopsis. *BMC Genomics* **7**, 327 (2006).

23. Haas, B. J. *et al.* Automated eukaryotic gene structure annotation using EVidenceModeler and the Program to Assemble Spliced Alignments. *Genome Biol.* **9**, R7 (2008).

24. Quevillon, E. *et al.* InterProScan: protein domains identifier. *Nucleic Acids Res.* **33**, W116–20 (2005).

25. Lyons, E., Pedersen, B., Kane, J. & Freeling, M. The Value of Nonmodel Genomes and an Example Using SynMap Within CoGe to Dissect the Hexaploidy that Predates the Rosids. *Trop. Plant Biol.* **1**, 181–190 (2008).

26. Tang, H. *et al.* Screening synteny blocks in pairwise genome comparisons through integer programming. *BMC Bioinformatics* **12**, 102 (2011).

27. Maere, S. *et al.* Modeling gene and genome duplications in eukaryotes. *Proc. Natl. Acad. Sci. U. S. A.* **102**, 5454–5459 (2005).

28. Team, R. C. & Others. R: A language and environment for statistical computing. (2013).

29. Wickham, H. *ggplot2: Elegant Graphics for Data Analysis*. (Springer, 2016).

Supplementary Tables

Supplementary Table 1: Genome assembly of *Fragaria iinumae*

Predicted Genome Size	265.56Mb
Predicted heterozygosity	0.18%
Illumina reads (250bp and 450bp)	28.42Gb
PacBio reads	45.77Gb
Total reads	74.19Gb
Total Sequence Coverage	279.37x
Genome Coverage > 4X	99.80%
Assembled Genome Size	240.58Mb
Total number contigs	94
Length of contig N50	10.67Mb
Number of contig N50	8
Length of contig N90	3.13Mb
Number of contig N90	22
GC content	39.70%
Anchored chromosome size	239.09Mb
Anchored chromosomes (%)	99.38%

Supplementary Table 2: Gene annotation of *Fragaria iinumae* genome

Gene numbers	23,665
BUSCO Assessment	94.80%
Average gene length (base pairs)	2,686.96
Average CDS length (base pairs)	1,254.27
Average exons per gene	5.09
Average exon length (base pairs)	246.48
Average intron length (base pairs)	350.4

Supplementary Table 3: Repeat element content in *F. iinumae* genome (number / percent of genome)

DNA transposon	12,405 / 5.16%
LINE retrotransposon	3,591 / 1.49%
SINE retrotransposon	12 / 0.005%
LTR retrotransposon	81,237 / 33.77%
Unclassified transposon	3,816 / 1.59%
Total transposable elements	99,805 / 41.49%
Total repeat elements	102,724 / 42.70%

Supplementary Table 4: Number of non-coding RNA genes in *F. iinumae* genome

miRNA	280
tRNA	461
rRNA	128
Small nuclear RNA	545
