# Supplementary material for: Reply to: Revisiting the origin of octoploid strawberry
Source: Nat Genet. 2019 Dec 16;52(1):5–7. doi: 10.1038/s41588-019-0544-2 (PMC6960091; doi:10.1038/s41588-019-0544-2)
Supplement: Supplementary file 2 — Reporting Summary [file 41588_2019_544_MOESM2_ESM.pdf]

nature research

Corresponding author(s): Patrick P. Edger, Qin Qiao, Ticao Zhang,

Last updated by author(s): October 29th, 2019

Reporting Summary

Nature Research wishes to improve the reproducibility of the work that we publish. This form provides structure for consistency and transparency in reporting. For further information on Nature Research policies, see Authors & Referees and the Editorial Policy Checklist.

Statistics

For all statistical analyses, confirm that the following items are present in the figure legend, table legend, main text, or Methods section.

n/a	Confirmed	
☐	☒	The exact sample size (*n*) for each experimental group/condition, given as a discrete number and unit of measurement
☐	☒	A statement on whether measurements were taken from distinct samples or whether the same sample was measured repeatedly
☒	☐	The statistical test(s) used AND whether they are one- or two-sided<br>*Only common tests should be described solely by name; describe more complex techniques in the Methods section.*
☒	☐	A description of all covariates tested
☒	☐	A description of any assumptions or corrections, such as tests of normality and adjustment for multiple comparisons
☐	☒	A full description of the statistical parameters including central tendency (e.g. means) or other basic estimates (e.g. regression coefficient) AND variation (e.g. standard deviation) or associated estimates of uncertainty (e.g. confidence intervals)
☒	☐	For null hypothesis testing, the test statistic (e.g. *F*, *t*, *r*) with confidence intervals, effect sizes, degrees of freedom and *P* value noted<br>*Give P values as exact values whenever suitable.*
☒	☐	For Bayesian analysis, information on the choice of priors and Markov chain Monte Carlo settings
☒	☐	For hierarchical and complex designs, identification of the appropriate level for tests and full reporting of outcomes
☒	☐	Estimates of effect sizes (e.g. Cohen's *d*, Pearson's *r*), indicating how they were calculated

Our web collection on statistics for biologists contains articles on many of the points above.

Software and code

Policy information about availability of computer code

Data collection	All commericial DNA and RNA sequencing platforms used in this study are fully described.
Data analysis	All software used in this study for data analysis is fully described including specifying versions used. All custom software developed for this study has already been deposited on Github with weblinks (e.g. PhyDS; (https://github.com/mrmckain/PhyDS/)).

For manuscripts utilizing custom algorithms or software that are central to the research but not yet described in published literature, software must be made available to editors/reviewers. We strongly encourage code deposition in a community repository (e.g. GitHub). See the Nature Research guidelines for submitting code & software for further information.

Data

Policy information about availability of data

All manuscripts must include a data availability statement. This statement should provide the following information, where applicable:

- Accession codes, unique identifiers, or web links for publicly available datasets
- A list of figures that have associated raw data
- A description of any restrictions on data availability

Raw sequencing data is available under accession code BioProjects PRJNA544784 and PRJNA508389 in the Sequence Read Archive on NCBI (https://www.ncbi.nlm.nih.gov/sra). Genome assemblies and annotations are on NCBI GenBank (https://www.ncbi.nlm.nih.gov/genome) under the same BioProjects and also available on the Genome Database for Rosaceae (https://www.rosaceae.org/).

Field-specific reporting

Please select the one below that is the best fit for your research. If you are not sure, read the appropriate sections before making your selection.

☒ Life sciences ☐ Behavioural & social sciences ☐ Ecological, evolutionary & environmental sciences

For a reference copy of the document with all sections, see nature.com/documents/nr-reporting-summary-flat.pdf

Life sciences study design

All studies must disclose on these points even when the disclosure is negative.

Sample size	Phylogenetic analyses were conducted of hundreds of orthologous genes. The genome of a single individual of Fragaria iinumae was sequenced.
Data exclusions	No data was excluded from any analysis, unless described in the manuscript.
Replication	Boostrap analyses were performed as described in the manuscript.
Randomization	Randomizations, included bootstrap analyses, to assess the level of support for each clade in every gene tree.
Blinding	A blinded-experiment is not possible for phylogenetic or genomic analyses.

Reporting for specific materials, systems and methods

We require information from authors about some types of materials, experimental systems and methods used in many studies. Here, indicate whether each material, system or method listed is relevant to your study. If you are not sure if a list item applies to your research, read the appropriate section before selecting a response.

Materials & experimental systems

n/a	Involved in the study	
☒	☐	Antibodies
☒	☐	Eukaryotic cell lines
☒	☐	Palaeontology
☒	☐	Animals and other organisms
☒	☐	Human research participants
☒	☐	Clinical data

Methods

n/a	Involved in the study	
☒	☐	ChIP-seq
☒	☐	Flow cytometry
☒	☐	MRI-based neuroimaging
